# Characterization of potentially health-promoting constituents in sea fennel (*Crithmum maritimum*) cultivated in the Conero Natural Park (Marche region, Central Italy)

**DOI:** 10.1080/13880209.2023.2224820

**Published:** 2023-07-06

**Authors:** Antonietta Maoloni, Teresa Pirker, Eva-Maria Pferschy-Wenzig, Lucia Aquilanti, Rudolf Bauer

**Affiliations:** a Dipartimento di Scienze Agrarie, Alimentari ed Ambientali, Università Politecnica delle Marche, Ancona, Italy; b Institute of Pharmaceutical Sciences, University of Graz, Graz, Austria

**Keywords:** Accelerated solvent extraction, chlorogenic acid, triterpene saponins, hydroxylated fatty acids, LC/MS

## Abstract

**Context:**

Sea fennel (*Crithmum maritimum* L. [Apiaceae]) is an aromatic herb rich in bioactive molecules, such as polyphenols, with potential positive effects on human health.

**Objective:**

This study aimed at the characterization of sea fennel secondary metabolites, focusing on the phenolic fraction.

**Materials and methods:**

Samples of whole sprouts, sole leaves and sole stems were subjected to accelerated solvent extraction with methanol, and the resulting extracts were analyzed by high‑performance thin‑layer chromatography, high-performance liquid chromatography, and liquid chromatography coupled with diode array detection and high-resolution mass spectrometry (LC-DAD-HRMS).

**Results:**

HPTLC and HPLC analyses of sea fennel extracts showed similar chromatographic profiles among the tested samples, and the prevalence of chlorogenic acid within the phenolic fraction was verified. Ten hydroxycinnamic acids, including neochlorogenic acid, chlorogenic acid, cryptochlorogenic acid, isochlorogenic acid B, isochlorogenic acid A and isochlorogenic acid C, 11 flavonoid glycosides, e.g., rutin, hyperoside, isoquercitrin, two triterpene saponins and two hydroxylated fatty acids, were detected and annotated *via* liquid chromatography coupled with diode array detection and high-resolution mass spectrometry.

**Discussion and conclusions:**

The use of accelerated solvent extraction and LC-DAD-HRMS for the characterization of sea fennel secondary metabolites allowed the annotation of seven compounds newly detected in sea fennel, including triterpene saponins and hydroxylated fatty acids.

## Introduction

Sea fennel (*Crithmum maritimum* L.) is an aromatic plant belonging to the Apiaceae family. The plant grows in coastal areas of Mediterranean and Black Sea and Atlantic Europe. It is rich in bioactive substances with nutritional and medicinal value (Alves-Silva et al. 2020). Its fleshy and succulent leaves are used for the preparation of cooked meals, salads, and pickles (Meot-Duros and Magné 2009; Generalić Mekinić et al. 2016). In folk medicine, they are applied as carminative, digestive, vermifuge, diuretic, depurative, anti-inflammatory, tonic, and antiscorbutic drug, as well as in the treatment of wounds and common cold (Atia et al. 2011; Zafeiropoulou et al. 2020). In recent years their ethyl acetate extracts showed activity against hepatocellular carcinoma (HCC) *in vitro* (Gnocchi et al. 2020), acting on the metabolic pathways (Gnocchi et al. 2021) and on the bioenergetic profile (Gnocchi et al. 2022) of HCC cells, and improving their sensitivity towards sorafenib, a commonly used chemotherapeutic drug (Gnocchi et al. 2023). Sea fennel is characterized by the presence of several bioactive constituents like vitamin C, essential fatty acids, essential oils, and polyphenols. Previous phytochemical studies revealed a high content of phenolic acids, mainly chlorogenic acids (Franke 1982; Cunsolo et al. 1993; Meot-Duros and Magné 2009; Generalić Mekinić et al. 2016, 2018; Pereira et al. 2017; Boutellaa et al. 2019; Najjaa et al. 2020).

Polyphenols represent an ubiquitous and large group of plant metabolites displaying key functions along their entire life cycle. In humans, they exhibit important physiological activities, mainly counteracting oxidative stress. They are therefore consided to be useful in the prevention of diabetes, cancer, cardiovascular diseases and neurological ailments (Han et al. 2007; Vrhovsek et al. 2012; Rodrigues et al. 2015).

Most of the studies dealing with sea fennel polyphenols have been performed using extracts produced by classical room temperature extraction (Nabet et al. 2017; Boutellaa et al. 2019; Zafeiropoulou et al. 2020; Souid et al. 2021), which was in some cases enhanced by the application of ultrasonic waves (ultrasound-assisted extraction, UAE) (Kumar et al. 2021; Martins-Noguerol et al. 2022), with methanol/water (Martins-Noguerol et al. 2022) or ethanol/water (Souid et al. 2021) mixtures. By the use of pressurized solvent, regularly at a pressure between 10 and 15 MPa, and high temperatures, regularly between 50 and 200 °C (Wang and Weller 2006), accelerated solvent extraction (ASE) is an advanced technique allowing a more efficient extraction of phenolic compounds than classical (Li et al. 2019) or ultrasound assisted methods (Pietrzak et al. 2014; Repajić et al. 2020).

Given these premises, the present study aimed at the characterization of the bioactive compounds of sea fennel with a focus on the polyphenolic fraction. Methanolic extracts from the whole sprouts, sole leaves and sole stems, obtained by accelerated solvent extraction, were analyzed by high‑performance thin‑layer chromatography (HPTLC), high-performance liquid chromatography (HPLC), and liquid chromatography coupled with diode array detection and high-resolution mass spectrometry (LC-DAD-HRMS) in order to identify the whole range of contained phenolic constituents.

## Materials and methods

### Test samples

Sea fennel cultivated in the Conero Natural Park, south of Ancona, Italy, was kindly supplied in June 2021 by a producer of sea fennel-based food preserves (Rinci S.r.l., Castelfidardo, Ancona, Italy). Fresh sea fennel sprouts (approximately 1 kg) were transported to the laboratory under cooled conditions (4 ± 2 °C), dried in a dehydrator (Captain Jerky 110, Klarstein, Berlin, Germany) at 30 °C, and stored in plastic bags under vacuum condition at room temperature (∼18–20 °C), prior to the analysis. Eight samples were prepared from the whole sprouts (S1, S2, S3, S4, S5, S6, S7, S8), one from the stems only (S9), and another one only from leaves (S10).

### Chemicals and reagents

Methanol (ROTIPURAN^®^ ≥ 99.9%, p.a., ACS, ISO), chloroform (≥99%, DAB, BP, pure), ethyl acetate (≥99.5%, Ph.Eur., pure), polyethylene glycol 4000, diphenylboric acid β-aminoethyl ester complex (natural product reagent A) (≥98%, p.a.), formic acid (ROTIPURAN^®^ ≥ 98%, p.a., ACS), *ortho*-phosphoric acid (85% ROTIPURAN^®^, p.a., ISO), luteolin (ROTICHROM^®^ 90%), hyperoside (ROTICHROM^®^ TLC), chlorogenic acid (ROTICHROM^®^ TLC), diosmin (purum), esculetin (purum CHR), (+)-catechin (pract CHR), (–)-quinic acid (purum CHR), (–)-epicatechin (∼95%), ferulic acid (≥99%), gallic acid (≥98%, p.a., ACS), hesperidin (CHR), protocatechuic acid (purum CHR), rutin (purum), syringic acid (purum CHR), kaempferol (ROTICHROM^®^ CHR) and quercetin (puriss. CHR) were purchased from Carl Roth GmbH (Karlsruhe, Germany).

Neochlorogenic acid (≥98.0%), cryptochlorogenic acid (≥98.0%), isochlorogenic acid A (≥98.0%), isochlorogenic acid B (≥98.0%) and isochlorogenic acid C (≥98.0%) were purchased from Chengdu Push Bio-technology Co., Ltd (Wuhou Science Park, Chengdu, China).

Methanol (HiPerSolv, CHROMANORM^®^), acetonitrile (HiPerSolv, CHROMANORM^®^), and water (HiPerSolv, CHROMANORM^®^) were purchased from VWR International SAS (Fontenay Sous Bois, France), while glacial acetic acid (100% anhydrous for analysis) was bought from Merck KGaA (Darmstadt, Germany).

The reference compounds rosmarinic acid, *p*-coumaric acid (purum ≥ 98%, HPLC) and quercetin-3-*O*-glucoside were purchased from PhytoLab GmbH and Co. KG (Vestenbergsgreuth, Germany), Thermo Fisher Scientific, (Waltham, MA, USA), and Extrasynthese (Genay, France), respectively. Quercitrin CRS (European Pharmacopoeia Reference Standard) was purchased from EDQM (Strasbourg, France).

### Accelerated solvent extraction

Dried sea fennel samples were ground using an analytical mill (A 11 basic, IKA^®^-Werke GmbH & Co. KG Staufen, Germany), mixed 4:1 (w/w) ratio with diatomaceous earth (Thermo Fisher Scientific, Waltham, MA, USA), and successively extracted with methanol by means of an accelerated solvent extractor (Dionex™ ASE™ 150, Thermo Fisher Scientific, Waltham, MA, USA). Extraction was performed setting the parameters as follows: heat time: 5 min; static time: 5 min; rinse volume: 40%; purge time: 60 s; cycles: 3; temperature: 68 °C. The extracts were dried under nitrogen flow and then stored at −20 °C until use.

### Sample preparation

The dried extracts were dissolved in methanol at a concentration of 10 mg/mL, sonicated for 5 min at room temperature in an ultrasonic bath (Transsonic T 460/H, Elma Schmidbauer GmbH, Singen, Germany) and centrifuged for 15 min at 13.000 rpm with a centrifuge (Biofuge^®^ pico, Heraeus, Hanau, Germany), to obtain a clear sample for further analyses. The reference compounds were dissolved in methanol at a concentration of 1 mg/mL. For high‑performance thin‑layer chromatography analysis, three separate solutions with a mix of reference compounds were prepared as follows: mix 1: esculetin, protocatechuic acid, gallic acid, and hyperoside; mix 2: ferulic acid, quercitrin, rosmarinic acid, quercetin-3-*O*-glucoside, and rutin; mix 3: kaempferol, quercetin, and chlorogenic acid.

### High‑performance thin‑layer chromatography

High‑performance thin‑layer chromatographic (HPTLC) analyses were performed using a CAMAG-HPTLC system (CAMAG Chemie‑Erzeugnisse und Adsorptionstechnik AG, Muttenz, Switzerland) operated with winCATS software (CAMAG). Aliquots of samples (10 μL) and mixed reference compound solutions (5 μL) were applied to HPTLC glass plates coated with silica gel 60 F_254_ (Merck KGaA, Darmstadt, Germany) by a CAMAG Automatic TLC Sampler ATS 4. Two HPTLC separations were performed using two different mobile phase systems. In the first analysis, the application length for all the samples was set at 7 mm and a mobile phase consisting of chloroform-glacial acetic acid-methanol-water (64:32:12:8) was employed (Wagner and Bladt 1996). While, in the second analysis, the application length for all the samples was set at 8 mm and a mobile phase consisting of ethyl acetate-formic acid-glacial acetic acid-water (100:11:11:26) was used (Wagner and Bladt 1996). HPTLC plates were developed in a CAMAG Automatic Developing Chamber ADC2 after 20 min equilibration with saturation pad and 5 min plate preconditioning to a final migration distance of 75 mm. After drying, the plates were derivatized with natural products-polyethylene glycol reagent (NP/PEG). The plates were visualized and photographed with a CAMAG TLC visualizer 2 after development and after derivatization at UV 254 and 366 nm, and at white light.

### Liquid chromatography‑diode array detection mass spectrometry

#### Fingerprint analyses and annotation of major compounds

Liquid chromatography‑diode array detection-high‑resolution mass spectrometry (LC-DAD-HRMS) analyses were performed using an Ultimate 3000 HPLC hyphenated with a Q Exactive™ hybrid quadrupole Orbitrap mass spectrometer (Thermo Fisher Scientific) in both HESI positive and negative mode. The separation was carried out on a Zorbax Extend-C18 column (3.5 μm, 4.6 mm × 150 mm, Agilent). The mobile phase consisted of water + 0.1% formic acid (A) and acetonitrile + 0.1% formic acid (B). A flow rate of 1.0 ml/min was applied and the gradient was set as follows: 0–4 min, 12% B in A; 4–5 min, 12%–20% B in A; 5–15 min, 20% B in A; 15–22 min, 20%–95% B in A; 22–24 min, 95% B in A; 24–25 min, 95%–12% B in A; 25–30 min, 12% B in A. The column temperature was set to 30 °C. The diode array detector was set to a range from 200 to 400 nm. The mass spectrometer was run in both, HESI positive and negative modes using the following parameters: probe heater temperature 350 °C; capillary temperature 330 °C; spray voltage 3.5 kV for positive and 3.1 kV for negative ion mode; sheath gas flow 65 arbitrary units; auxiliary gas flow 20 arbitrary units; resolution: 70.000 (full MS) and 17.500 (data‑dependent MS^2^). A volume of 5 μL was injected for the samples, reference and blank solutions. Data evaluation was performed with Thermo Xcalibur 2.2.44 (Thermo Fisher Scientific). Compounds were annotated by comparing retention time, precursor monoisotopic mass, and MS/MS fragment ion masses with authentic references, or by comparing MS/MS fragmentation patterns with literature. Molecular formulas were calculated from the exact mass using Thermo Xcalibur 2.2.44 software (Thermo Fisher Scientific).

#### Annotation of flavonoid aglycone moieties, triterpene saponins and hydroxylated fatty acids by LC-MS^n^

Identification of flavonoid aglycones was performed by an Ultimate 3000 HPLC hyphenated LTQ-XL linear ion trap mass spectrometer with HESI interface (Thermo Fisher Scientific) operated in negative mode. The HPLC separation was carried out as described in the section *'Fingerprint analyses and annotation of major compounds’*. The mass spectrometer was run in the HESI negative mode using the following parameters: source heater temperature 350 °C; capillary temperature 330 °C; spray voltage 3.0 kV, sheath gas flow 50 arbitrary units; auxiliary gas flow 10 arbitrary units. The volume of 5 μL was injected for the samples, for reference and blank solutions. Flavonoid aglycone moieties were identified by comparison of the MS^3^ or MS^4^ fragmentation patterns in the respective glycosides to MS^2^ fragmentation patterns of authentic reference compounds of the respective aglycones. Additionally, these data were used for annotation of compounds **23**–**26**.

### Semiquantitative determination of hydroxycinnamic acid derivative and flavonoid levels by high performance liquid chromatography with diode array detection

High performance liquid chromatography (HPLC) analyses were performed by a 1260 Infinity HPLC-DAD system (Agilent Technologies, Inc., Santa Clara, CA, USA). Separation was performed with the same system described in the section *'Fingerprint analyses and annotation of major compounds’*, with the exception that the mobile phase consisted of water + 0.1% ortho-phosphoric acid (A) and acetonitrile (B). DAD-detection was carried out at 320 and 360 nm. Chlorogenic acid and hydroxycinnamic acid derivatives were quantified using chlorogenic acid as external standard, while flavonoids were quantified using rutin as external standard. Calibration curves were prepared using six different concentrations of the reference compounds dissolved in methanol (1, 10, 50, 100, 500, 1000 μg/mL) and were injected in the same condition as the samples. For quantification, the peak area of each hydroxycinnamic acid derivative was recorded at 320 nm. For establishing the rutin calibration curve, detection was carried out at 360 nm. The results were calculated as g/100 g dry weight (DW) of sea fennel, and expressed as mean value of two replicates ± standard deviation. Chlorogenic acid concentration was calculated from the peak area of peak 3. The level of total hydroxycinnamic acid derivatives was calculated using the areas of peaks 2, 3, 4, 6, 7, 8, 9, 15, 16 and 18. The approximate flavonoid content was calculated from the areas of peaks 5, 10, 11, 12, 13, 14, 17, 19, 20, 21 and 22 (peak numbers as in Table 2). Data evaluation was performed with Agilent ChemStation.

### Statistical analysis

The results of the quantification of chlorogenic acid, hydroxycinnamic acid derivatives and flavonoids were subjected to one-way analysis of variance (ANOVA) through the Tukey-Kramer honest significant difference (HSD) test (*p* ≤ 0.05), to evaluate differences between the samples. The software JMP Version 11.0.0 (SAS Institute Inc., Cary, NC, USA) was used for the analysis.

## Results

### Accelerated solvent extraction

The extraction yields of each sample are listed in Table 1, as absolute (g) and relative (%) values. Stems (sample S9) provided higher extraction yields (19.01%) than leaves (sample S10, 14.30%).

**Table 1.** Absolute and relative extract yields obtained by accelerated solvent extraction (ASE) of sea fennel whole sprouts (sample S1, S2, S2, S3, S4, S5, S6, S7, S8), stems (S9) and leaves (S10).

Sample	Initial weight (g)	Yield MeOH extract (g)	Yield MeOH extract (%)
S1	8.79	1.37	15.55
S2	7.70	1.46	18.98
S3	9.30	1.70	18.25
S4	7.81	1.47	18.80
S5	10.08	1.76	17.51
S6	9.88	1.62	16.35
S7	11.28	1.89	16.79
S8	10.98	1.65	15.07
S9	8.99	1.71	19.01
S10	11.23	1.61	14.30

### Chemical profiling by high‑performance thin‑layer chromatography

**Figure 1.**
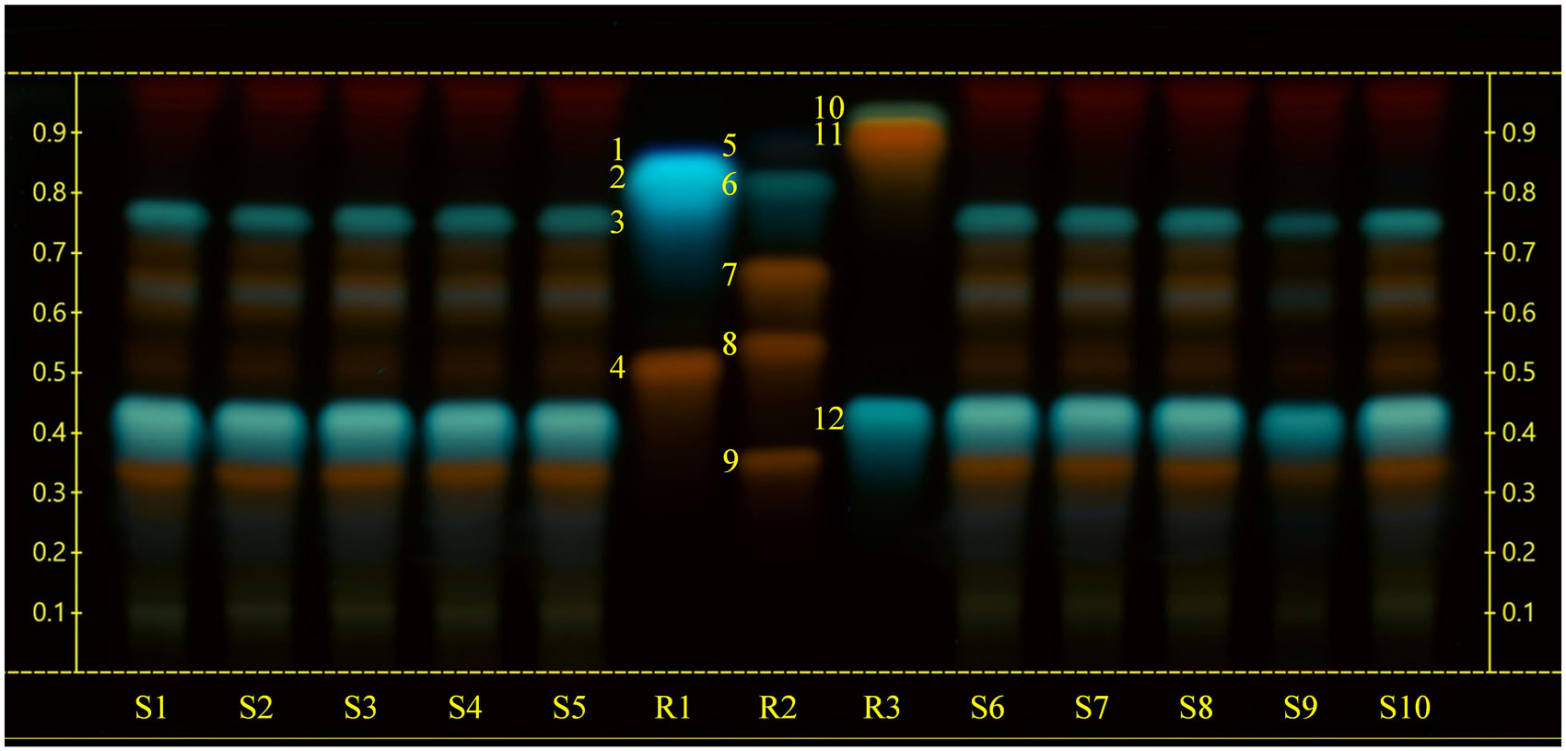
High‑performance thin‑layer chromatography (HPTLC) carried out using a mobile phase consisting of ethyl acetate-formic acid-glacial acetic acid-water (100:11:11:26), derivatized with natural products-polyethylene glycol reagent (NP/PEG) and observed at UV 366 nm. S1, S2, S3, S4, S5, S6, S7, S8: sea fennel whole sprouts; S9 sea fennel stems; S10: sea fennel leaves. R1: reference compounds mix 1 (**1**: protocatechuic acid; **2**: esculetin; **3**: gallic acid; **4**: hyperoside); R2: reference compounds mix 2 (**5**: ferulic acid; **6**: rosmarinic acid; **7**: quercitrin; **8**: quercetin-3-*O*-glucoside; **9**: rutin); R3: reference compounds mix 3 (**10**: kaempferol; **11**: quercetin; **12**: chlorogenic acid).

The HPTLC analyses of sea fennel extracts highlighted similar chromatographic profiles among the tested samples. The use of a mobile phase generally employed for the analysis of polyphenols, namely ethyl acetate-formic acid-glacial acetic acid-water (100:11:11:26), allowed a proper chromatographic separation of rutin, chlorogenic acid, hyperoside, quercetin-3-*O*-glucoside, quercitrin, and rosmarinic acid, while the use of chloroform-glacial acetic acid-methanol-water (64:32:12:8) as mobile phase allowed the separation of gallic acid, protocatechuic acid, esculetin, quercetin, kaempferol and ferulic acid. By the use of corresponding reference compounds and derivatization with natural products-polyethylene glycol reagent (NP/PEG) (Figure 1) we could identify rutin (*R*_f_ = 0.34) and chlorogenic acid (*R*_f_ = 0.42).

**Figure 2.**
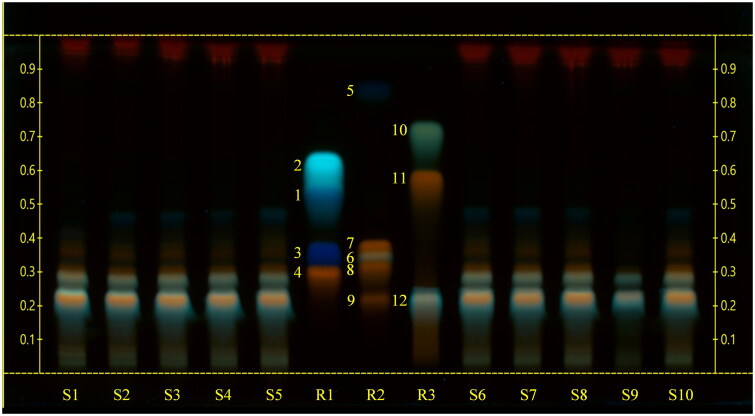
High‑performance thin‑layer chromatography (HPTLC) carried out using a mobile phase consisting of chloroform-glacial acetic acid-methanol-water (64:32:12:8), derivatized with natural products-polyethylene glycol reagent (NP/PEG) and observed at UV 366 nm. S1, S2, S3, S4, S5, S6, S7, S8: sea fennel whole sprouts; S9 sea fennel stems; S10: sea fennel leaves. R1: reference compounds mix 1 (**1**: protocatechuic acid; **2**: esculetin; **3**: gallic acid; **4**: hyperoside); R2: reference compounds mix 2 (**5**: ferulic acid; **6**: rosmarinic acid; **7**: quercitrin; **8**: quercetin-3-*O*-glucoside; **9**: rutin); R3: reference compounds mix 3 (**10**: kaempferol; **11**: quercetin; **12**: chlorogenic acid).

Three more compounds, namely hyperoside, quercetin-3-*O*-glucoside and quercitrin were tentatively allocated, but their bands in the samples were not well enough separated for unambiguous identification. Ferulic acid, kaempferol, quercetin, esculetin, protocatechuic acid, gallic acid and rosmarinic acid could not be detected in the extracts by HPTLC (Figure 2).

As far as the intensity of the bands was concerned, chlorogenic acid was the predominant compound in all the samples.

### Chemical profiling of whole sea fennel sprouts methanolic extract by liquid chromatography‑diode array detection mass spectrometry

Preliminary HPLC analyses, performed as described in the section *'Semiquantitative determination of hydroxycinnamic acid derivative and flavonoid levels by high performance liquid chromatography with diode array detection’*, had confirmed the presence of the same compounds in all the test samples (data not shown). Therefore, the sample S1, constituted by sea fennel whole sprouts, was chosen as representative for LC-DAD-HRMS analyses.

**Figure 3.**
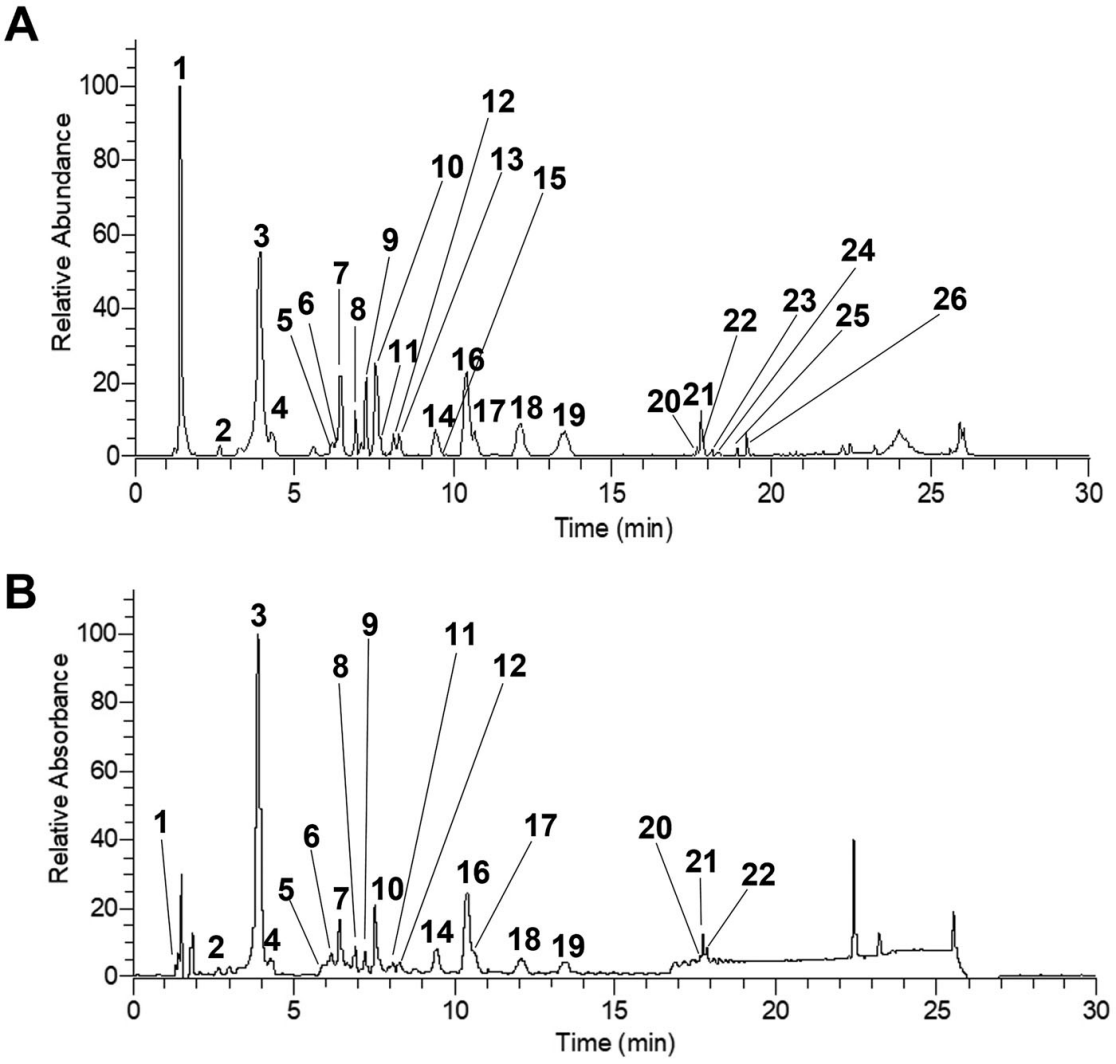
Base peak chromatogram of sea fennel methanolic extract in HESI negative mode full MS [*m/z* 110–1650] (A) and DAD (200–400 nm) (B).

The chromatograms generated from sample S1 by HESI negative mode and DAD detection are depicted in Figure 3.

HESI negative mode was used for compound annotation because more compounds were detectable in the negative than in the positive mode. In sea fennel methanolic extracts, 26 compounds could be annotated, belonging to the following classes: organic acids, hydroxycinnamic acid derivatives, flavones, flavonols, triterpene saponins, and hydroxylated fatty acids (Table 2).

**Table 2.** Constituents annotated from sample S1, ordered by retention time (see Figure 3).

Compound No.	RT (min)	DAD λ_max_ (nm)	Negative ion mode HRMS data					Annotation		
			*m/z* parent ion	*m/z* MS/MS fragment ions	Neutral monoisotopic mass (Da)	Calculated molecular formula	Δ (ppm)	Identity	Compound class	Literature reference
1	1.44		191.0563 [M-H]^–^	85.0296 (7), **191.0563 (100)**	192.0634	C_7_H_12_O_6_	4.582	quinic acid*	organic acid	
2	2.62	215, 325	353.0881 [M-H]^–^	135.0452 (32), 179.0350 (71), **191.0563 (100)**, 353.0881 (24)	354.0951	C_16_H_18_O_9_	2.751	neochlorogenic acid*	hydroxycinnamic acid	
3	3.92	215, 240, 325	353.0874 [M-H]^–^	**191.0562 (100)**	354.0951	C_16_H_18_O_9_	2.043	chlorogenic acid*	hydroxycinnamic acid	
4	4.31	220, 240, 325	353.0882 [M-H]^–^	135.0453 (36), **173.0456 (100)**, 179.0351 (71), 191.0564 (98), 353.0880 (21)	354.0951	C_16_H_18_O_9_	2.383	cryptochlorogenic acid*	hydroxycinnamic acid	
5	6.19	210, 275, 325	593.1522 [M-H]^–^	353.1528 (76), 383.0779 (46), 473.1093 (31), 503.1209 (8), **593.1525 (100)**	594.1585	C_27_H_30_O_15_	1.945	vicenin 2	flavone-C-glycoside	(Gouvea et al. 2017; Zafeiropoulou et al. 2020)
6	6.33	210, 275, 325	353.0876 [M-H]^–^	**191.0564 (100)**	354.0951	C_16_H_18_O_9_	2.383	cis-5-caffeoylquinic acid	hydroxycinnamic acids	(Clifford et al. 2008; Mhlongo et al. 2015)
7	6.47	215, 310	337.0927 [M-H]^–^	93.0348 (15), 163.0404 (9), 173.0458 (10), **191.0565 (100)**, 337.0937 (6)	338.1002	C_16_H_18_O_8_	2.718	trans-5-*O*-p-coumaroylquinic acid	hydroxycinnamic acids	(Jaiswal et al. 2014; Granato et al. 2016; Sánchez-Faure et al. 2020)
8	6.91	220, 240, 325	367.1031 [M-H]^–^	93.0347 (25), 173.0456 (45), **191.0564 (100)**, 193.0506 (14), 367.1038 (9)	368.1107	C_17_H_20_O_9_	2.074	5-*O*-feruoylquinic acid	hydroxycinnamic acids	(Jaiswal et al. 2014; Sánchez-Faure et al. 2020)
9	7.29	235, 305	337.0927 [M-H]^–^	**191.0562 (100)**, 192.0598 (8), 337.0934 (5)	338.1002	C_16_H_18_O_8_	2.629	cis-5-*O*-p-coumaroylquinic acid	hydroxycinnamic acids	(Jaiswal et al. 2014; Nabet et al. 2017; Piatti et al. 2023)
10	7.57	205, 255, 355	609.1472 [M-H]^–^	271.0252 (9), **300.0278 (100)**, 301.0342 (40), 609.1474 (58)	610.1534	C_27_H_30_O_16_	1.722	rutin*	flavonol-*O*-glycoside	
11	7.68	255, 350	593.1514 [M-H]^–^	96.9601 (4) 178.9630 (3), **285.0406 (100)**, 593.1526 (40),	594.1585	C_27_H_30_O_15_	2.147	luteolin- 7-*O*-hexosyl-deoxyhexoside	flavone-*O*-glycoside	
12	8.13	255, 350	463.0890 [M-H]^–^	271.0253 (11), **300.0279 (100)**, 301.0346 (59), 463.0890 (51)	464.0955	C_21_H_20_O_12_	2.090	hyperoside*	flavonol-*O*-glycoside	
13	8.28	255, 350	463.0882 [M-H]^–^	255.0298 (6), 271.0250 (12), **300.0279 (100)**, 301.0346 (55), 463.0890 (49)	464.0955	C_21_H_20_O_12_	2.305	isoquercitrin*	flavonol-*O*-glycoside	
14	9.45	250, 330	433.0773 [M-H]^–^	271.0249 (10), **300.0278 (100)**, 301.0338 (35), 433.0781 (42)	434.0849	C_20_H_18_O_11_	1.829	quercetin-3-*O*-pentoside	flavonol-*O*-glycoside	(Sánchez-Faure et al. 2020)
15	9.52	250, 325	515.1192 [M-H]^–^	135.0453 (27), 161.0245 (20), **173.0455 (100)**, 179.0351 (83), 191.0564 (45), 335.0780 (13), 353.0882 (43), 515.1199 (36)	516.1268	C_25_H_24_O_12_	1.548	isochlorogenic acid B*	hydroxycinnamic acids	
16	10.42	220, 240, 325	515.1189 [M-H]^–^	135.0453 (18), 179.0350 (70), 173.0455 (5), **191.0563 (100)**, 353.0882 (60)	516.1268	C_25_H_24_O_12_	0.966	isochlorogenic acid A*	hydroxycinnamic acids	
17	10.68	215, 250, 335	607.1672 [M-H]^–^	284.0328 (32), **299.0563 (100)**, 300.0598 (17), 607.1674(7)	608.1741	C_28_H_32_O_15_	2.460	diosmin*	flavone-*O*-glycoside	(Adouani et al. 2013; Sánchez-Faure et al. 2020)
18	12.10	245, 325	515.1199 [M-H]^–^	135.0453 (19), **173.0455 (100)**, 179.0350 (80), 191.0563 (32), 353.0882 (71), 515.1199 (8)	516.1268	C_25_H_24_O_12_	1.432	isochlorogenic acid C*	hydroxycinnamic acid	
19	13.49	255, 330	625.1214 [M-H]^–^	151.0037 (7), 161.0244 (7), 178.9988 (6), 271.0240 (5), **300.0278 (100**), 301.0351 (80), 463.0888 (60), 625.1215 (29)	626.1272	C_30_H_26_O_15_	2.597	quercetin-3-*O*-caffeoylhexoside	flavonol-*O*-hydroxycinnamoylglycoside	(Farag et al. 2015)
20	17.67	215, 315	609.1256 [M-H]^–^	151.0037 (6), 178.9987 (5), 255.0301 (6), 271.0251 (9), **300.0278 (100)**, 301.0348 (60), 463.0888 (40), 609.1261 (38)	610.1323	C_30_H_26_O_14_	2.739	quercetin-3-*O*-p-coumaroylhexoside isomer 1	flavonol-*O*-hycroxycinnamoylglycoside	(García-Villalba et al. 2017; Zafeiropoulou et al. 2020)
21	17.76	215, 260, 315	609.1252 [M-H]^–^	151.0038 (5), 178.9999 (3), 255.0303 (6), 271.0249 (8), **300.0278 (100)**, 301.0345 (45), 463.0889 (37), 609.1264 (34)	610.1323	C_30_H_26_O_14_	2.131	quercetin-3-*O*-p-coumaroylhexoside isomer 2	flavonol-*O*-hycroxycinnamoylglycoside	(García-Villalba et al. 2017; Zafeiropoulou et al. 2020)
22	17.87	215, 330	639.1367 [M-H]^–^	151.0034 (3), 178.9982 (2), 255.0305 (6), 271.0250 (9), **300.0279 (100)**, 301.0342 (43), 463.0885 (27), 639.1372 (32)	640.1428	C_31_H_28_O_15_	3.463	quercetin-3-*O*-feruloylhexoside	flavonol-*O*-hydroxycinnamoylglycoside	(De Andrade Neves et al. 2018)
23	18.16		1147.5604 [M-H]^–^	221.0669 (32), 441.3380 (6), 451.3218 (6), 521.3641 (32), 553.3907 (18), 953.4830 (6), 967.4951 (7), 985.5064 (90),, **1147.5609 (100**)	1148.5615	C_55_H_88_O_25_	6.384	trihexosyl-monohexuronyl triterpene (aglycone: C_31_H_50_O_4_)	triterpene saponin	
24	18.24		1115.5343 [M-H]^–^	221.0667 (25), 453.3375 (5), 521.3638 (48), 567.3706 (12), 611.3591 (5), 953.4800 (17), **1115.5343 (100)**	1116.5353	C_54_H_84_O_24_	6.652	trihexosyl-monohexuronyl triterpene (aglycone: C_30_H46O_3_)	triterpene saponin	
25	18.91		327.2177 [M-H]^–^	171.1026 (12), 183.1388 (9), 211.1339 (32), 229.1445 (19), 291.1965 (7), 309.2090 (3), **327.2180 (100)**	328.2250	C_18_H_32_O_5_	3.482	trihydroxy-octadecadienoic acid	hydroxylated fatty acid	(Farag et al. 2015)
26	19.21		329.2333 [M-H]^–^	139.1130 (10), 157.1234 (5), 171.1028.(33), 183.1395 (5), 211.1343.(32), 229.1447 (18), **329.2338 (100)**	330.2406	C_18_H_34_O_5_	3.309	tianshic acid or isomer	hydroxylated fatty acid	(Gao et al. 2016; Kothari et al. 2020)

Compounds marked with * were identified by comparison with authentic reference compound (MSI level 1), the other compounds by comparison with literature data (MSI level 2) (Sumner et al. 2007) or by theoretical interpretation of MS/MS spectra (MSI level 3). MS/MS base peak is printed in bold.

Eleven of these compounds were unambiguously identified by comparing their retention times, precursor monoisotopic mass and MS/MS fragmentation patterns with that of authentic reference substances, and 15 compounds were tentatively annotated by comparing precursor monoisotopic mass, MS/MS fragmentation patterns and the molecular formulas calculated from the exact mass with existing data from literature. In cases where MS/MS fragmentation was not sufficient for annotation, MS^3^ and MS^4^ fragmentation patterns generated in a linear ion trap mass spectrometer were additionally used.

Ten hydroxycinnamic acids were detected in the extract, six of them were unambiguously identified as neochlorogenic acid (**2**), chlorogenic acid (**3**), cryptochlorogenic acid (**4**), isochlorogenic acid B (**15**), isochlorogenic acid A (**16**) and isochlorogenic acid C (**18**).

The fragmentation pattern of compound **6** indicated that it is composed of a caffeic acid and a quinic acid moiety. The MS/MS fragmentation pattern is the same as 5-caffeoylquinc acid (chlorogenic acid; compound **3**) but it eluted significantly later. Accordingly, it was assigned as *cis*-5-caffeoylquinic acid (Clifford et al. 2005, 2008).

The isomeric compounds **7** and **9** exhibited a MS/MS fragment with *m/z* 191, matching quinic acid, that was obviously generated by the neutral loss of a coumaroyl moiety. The MS/MS base peak at *m/z* 191 indicated a 5-*O*-coumaroylquinic acid (Clifford et al. 2003). According to retention times, compounds **7** and **9** were annotated as trans-5-*O*-p-coumaroylquinic acid and cis-5-*O*-p-coumaroylquinic acid, respectively (Jaiswal et al. 2014; Nabet et al. 2017).

Compound **8** also exhibited a MS/MS fragment at *m/z* 191 [M-H-C_10_H_8_O_3_]^–^. The neutral loss of C_10_H_8_O_3_ indicated a feruloyl moiety that was obviously attached to quinic acid. The MS/MS base peak at *m/z* 191 indicated that the ferulic acid moiety is attached at position 5 (Clifford et al. 2003). Hence the compound was annotated as 5-*O*-feruoylquinic acid.

In total, 11 flavonoid glycosides were annotated: three flavone and eight flavonol glycosides. Flavones were represented by two *O*-glycosides and one C-glycoside. Among *O*-glycosidic flavones, compound **11** generated a parent ion at *m/z* 593.1514 [M-H]^–^, corresponding to the calculated molecular formula C_27_H_30_O_15_. The fragment at *m/z* 447 was caused by the loss of the deoxyhexose, and the combined loss of a hexose and a deoxyhexose moiety caused the fragment at *m/z* 285 [M-H-hexose-deoxyhexose]^–^. MS^n^ fragmentation patterns indicated a 7-*O*-diglycoside moiety (Cuyckens and Claeys 2004; Vukics and Guttman 2010). The aglycone was annotated as luteolin by comparison with an authentic luteolin reference (Table 3). Accordingly, compound **11** was tentatively annotated as luteolin-7-*O*-hexosyl-deoxyhexoside.

**Table 3.** MS^n^ fragmentation data of selected compounds.

Compound No.	Negative ion mode MS data			
	Parent ion	MS^2^ fragments	MS^3^ fragments	MS^4^ fragments
11	593.2	**285.0 (100),** 447.2 (2)	285.0 (30), 257.0 (24), **241.1 (100),** 217.0 (57), 212.8 (22), 198.8 (58), 175.0 (69), 151.0 (24),	–
14	433.2	301.0	283.0 (12), 273.0 (14), 271.1 (13), 257.0 (15), 255.0 (11), **179.0 (100),** 151.0 (70)	–
19	625.1	463.1 (100), 301.1 (18)	301.1	273.2 (13), 257.1 (14), **178.9 (100),** 150.9 (62)
20	609.2	463.1 (100), 301.1 (11)	301.1	273.1 (12), 257.1 (16), 239.1 (14) **179.0 (100),** 150.8 (83)
21	609.2	463.1 (100), 301.0 (13)	301.1	273.1 (14), 257.1 (15), **178.9 (100),** 150.9022 (62)
22	639.2	477.2 (23), 463.2 (100), 301.1 (17)	301.1	272.9 (13), 257.1 (21), **179.0 (100),** 150.9 (72)
23	1147.5	985.5	967.6 (30), 953.5 (23), 909.6 (28), 823.4 (13), 805.5 (3), 643.5 (8), 625.4 (54), **553.5 (100),** 521.5 (11), 483.5 (12), 477.5 (8), 451.4 (10)	485.4 (53**), 483.5 (100)**, 451.4 (32)
24	1115.6	1097.5 (10), **953.5 (100),** 521.3 (28)	935.2 (26), 791.2 (14), 611.4 (18), 593.4 (19), **521.5 (100)**, 453.5 (11)	491.4 (34), 477.6 (17), **453.4 (100)**
25	327.2	309.3 (35), 293.1 (59), **229.2 (100),** 211.1 (33), 171.0 (40)	**211.1 (100**), 209.2 (33), 183.1 (9), 171.0 (9), 155.1 (23), 127.1 (29), 124.9 (16)	–
26	329.3	311.2 (22), 293.2 (38), **229.1.(100**), 211.1 (52), 171.0 (16),	**211.2 (100),** 167.1 (14), 155.1 (13), 125.0 (22)	193.1 (35), **183.1 (100),** 167.0 (61), 125.1 (30).
luteolin*	285.1	285.0 (30), 257.0 (18) **240.9 (100),** 217.0 (59), 212.9998 (20) 198.9 (53), 174.9 (75) 151.0 (26)	–	–
quercetin*	301.0	273.1 (15), 257.0 (14), **179.0 (100),** 150.9 (68)	–	–

MS^n^ base peak is printed in bold. *Authentic reference.

Compound **17** presented a [M-H]^–^ ion at *m/z* 607.1672, corresponding to the molecular formula C_28_H_32_O_15_.The fragment at *m/z* 299 derived from the combined loss of a hexose and a deoxyhexose. A neutral loss of 15 Da indicates the presence of a cleavable methyl group within the aglycone. By comparison with an authentic reference, compound **17** was assigned as diosmin (diosmetin 7-*O*-rutinoside), previously reported in sea fennel (Cornara et al. 2009).

Compound **5** showed a parent ion at *m/z* 593.1513 [M-H]^–^, corresponding to a calculated neutral formula of C_27_H_30_O_15_. Fragment ions at *m/z* 503 [M-H-90]^–^, 473 [M-H-120]^–^, 383 [M-H-90-120]^–^, 353 [M-120-120]^–^ indicated the presence of two C-glycosidic hexose moieties (Cuyckens and Claeys 2004). By comparison with literature data, compound **5** was assigned vicenin 2 (apigenin-6,8-di-C-glycopyranoside), previously reported from sea fennel (Gouvea et al. 2017; Zafeiropoulou et al. 2020).

All annotated flavonols were *O*-glycosides, including rutin, (**10**), hyperoside (**12**) and isoquercitrin (**13**), which were unambiguously identified by comparison of their retention times and MS/MS fragmentation patterns with reference standards.

The parent ion of compound **14** was detected at *m/z* 433.0773 [M-H]^–^. The neutral loss of 132 Da indicated the presence of an *O*-glycosidic pentose moiety. By comparison of its MS^3^ fragmentation pattern with the MS^2^ fragmentation patterns of an authentic flavonoid aglycone reference, the aglycone was identified as quercetin (Table 3). Additionally, the fragment at *m/z* 271 [Aglycone-H-2H-CO]^–^ indicated that the pentose moiety was attached at position 3 (Vukics and Guttman 2010). Hence, compound **14** was tentatively annotated as quercetin-3-*O*-pentoside.

Beside flavonol monoglycosides, four flavonol-hydroxycinnamoyl-glycosides were annotated. Compounds **20** and **21** both exhibited parent ions at *m/z* 609.1256 and a calculated molecular formula of C_30_H_26_O_14_. Both compounds exhibited an MS/MS fragment ion at *m/z* 463. Neutral loss of 146 Da and calculated molecular formula of the neutral fragment (C_9_H_6_O_2_) indicated the cleavage of a coumaroyl moiety. Subsequent loss of 162 Da and the absence of a fragment at *m/z* 447 [M-Hex-H]^–^ led to the conclusion that both compounds contain *O*-coumarylhexose moieties (Kachlicki et al. 2016). In analogy to compound **14**, the aglycone was assigned to quercetin and the fragment at 271 indicated glycosylation in position 3, leading to the assignment of compound **20** and **21** to two isomeric quercetin-3-*O*-coumaroylhexosides.

Similarly, compound **19** and **22** consisted of quercetin and an *O*-glycosidic hexose. In these cases, calculated molecular formulas and neutral losses of C_9_H_6_O_3_ and C_10_H_8_O_3_ led to the assumption that compounds **19** and **22** contained a caffeic acid and a ferulic acid moiety, respectively. Accordingly, compound **19** was annotated as quercetin-3-*O*-caffeoyl-hexoside and compound **22** as quercetin-3-*O*-feruloylhexoside.

Two triterpene saponins were also annotated. MS/MS fragmentation patterns of both, compounds **23** and **24**, indicated the presence of three hexose and a glucuronic acid moiety. The aglycones of compounds **23** and **24** were found to possess calculated molecular formulas of C_31_H_50_O_4_ and C_30_H_46_O_3_, respectively.

Finally, two hydroxylated fatty acids were annotated. The calculated molecular formula (C_18_H_32_O_5_) and the fragmentation pattern of compound **25** indicated a trihydroxy-octadecadienoic acid (FA 18:2; O3). The calculated molecular formula (C_18_H_34_O_5_) and the fragmentation pattern of compound **26** indicated a trihydroxy-octadecenoic acid (FA 18:1; O3). By comparing MS/MS data of **26** with literature, the compound was annotated tianshic acid (Gao et al. 2016; Kothari et al. 2020).

### Semiquantitative determination of hydroxycinnamic acid derivative and flavonoid levels

The results of the semiquantitative determination of hydroxycinnamic acid derivatives and flavonoids are reported in Table 4.

**Table 4.** Concentration of chlorogenic acid, hydroxycinnamic acid derivatives and flavonoids in sea fennel.

Sample	Chlorogenic acid (g/100g DW)	Hydroxycinnamic acid derivatives (g/100g DW)	Flavonoids (g/100g DW)
S1	0.81 ± 0.02^d^	1.29 ± 0.03^f^	0.29 ± 0.00^f^
S2	1.03 ± 0.01^b^	1.59 ± 0.02^b^	0.44 ± 0.00^bc^
S3	1.19 ± 0.00^a^	1.87 ± 0.01^a^	0.46 ± 0.00^a^
S4	1.02 ± 0.00^b^	1.54 ± 0.00^bc^	0.38 ± 0.00^e^
S5	0.93 ± 0.00^c^	1.46 ± 0.00^de^	0.46 ± 0.00^ab^
S6	1.01 ± 0.00^b^	1.57 ± 0.01^b^	0.39 ± 0.01^de^
S7	0.95 ± 0.01^c^	1.51 ± 0.02^cd^	0.43 ± 0.00^c^
S8	0.83 ± 0.01^d^	1.34 ± 0.01^f^	0.40 ± 0.01^d^
S9	0.43 ± 0.00^e^	0.77 ± 0.00^g^	0.15 ± 0.00^g^
S10	0.93 ± 0.01^c^	1.43 ± 0.01^e^	0.44 ± 0.00^c^

Values are expressed as mean value ± standard deviation as g/100g DW sea fennel. Values labelled with different letters in the same column are significantly different (*p* < 0.05).

One-way analysis of variance (ANOVA) highlighted significant differences in flavonoid and hydroxycinnamic acid derivative levels among the whole sprout samples. All the compounds resulted to be less concentrated in stems (S9) than in leaves (S10). Whereas, chlorogenic acid resulted to be the most abundant polyphenol with contents ranging from 0.81 (S1) to 1.19 (S3) g/100 g DW sea fennel in the whole sprouts.

## Discussion

Starting from the premise that sea fennel is rich in several classes of bioactive compounds and taking into consideration the increasing interest in polyphenols for their health benefits (Vrhovsek et al. 2012), this study aimed at a holistic characterization of sea fennel phenolic constituents. Accelerated solvent extraction using pure methanol as solvent was performed, in order to recover the phenolic compound-enriched fraction (Alonso-Salces et al. 2001; Sun et al. 2012) from the whole sprouts, leaves, and stems, to be subjected to further characterization.

The extraction yield is a parameter influenced not only by the extraction solvent and method, but also by the vegetable matrix (Pferschy-Wenzig and Bauer 2015). This assumption is in agreement with the different extraction yields obtained between leaves and stems.

The combination of two HPTLC methods with different mobile phases allowed a good separation for all the reference compounds of different polarity (Jesionek et al. 2015). The derivatization of the plates with natural products-polyethylene glycol reagent (NP/PEG), regularly employed for polyphenol analysis, and the observation at UV 366 nm returned structure-dependent typical fluorescence (Wagner and Bladt 1996).

Nineteen of the compounds annotated in sample S1 on the basis of LC-HRMS data have been detected in sea fennel in previous phytochemical studies (Nabet et al. 2017; Alves-Silva et al. 2020; Sánchez-Faure et al. 2020; Souid et al. 2020; Zafeiropoulou et al. 2020; Piatti et al. 2023), while seven compounds, namely luteolin-7-*O*-hexosyl-deoxyhexoside, quercetin-caffeoyl-hexoside, quercetin-feruloyl-hexoside, the two hydroxylated fatty acids and the two triterpene glycosides, were newly found in sea fennel.

Saponins are a group of compounds consisting of a triterpene or steroid aglycone and one or more sugar chains. These compounds, traditionally considered as ‘anti-nutritional factors’ in food, are recognized, nowadays, as the active principles in many herbs used in traditional medicine. Saponins exhibit a wide range of biological activities behaving as hypocholesterolemic, anti-mutagenic, anti-inflammatory, antioxidant, immunomodulatory, hepatoprotective and neuroprotective agents (Liu and Henkel 2002; Sparg et al. 2004; Güçlü-Üstündağ and Mazza 2007). Furthermore, among the tentatively identified compounds new for sea fennel, hydroxylated fatty acids are described in literature as bioactive compounds with antimicrobial, cytotoxic and anti-neuroinflammatory properties (Masoodi et al. 2008; Serag et al. 2020). Therefore, comprehensive analysis of sea fennel extract by LC-HRMS provided evidence that next to phenolic constituents, other potentially bioactive compounds like triterpene glycosides and hydroxy fatty acids are present that may deserve more in-depth evaluation.

As far as the semiquantitative determination of hydroxycinnamic acid derivatives and flavonoids is concerned, the lower concentration detected in stems than in leaves and the presence of chlorogenic acid as the most concentrated polyphenol are in agreement with previous studies by Pereira et al. (2017) and Meot-Duros and Magné (2009), respectively. Furthermore, the results herein collected agree with available data related to sea fennel growing in different geographical areas. In more detail, concentrations of chlorogenic acid of 0.64 g/100 g DW (Nabet et al. 2017), 0.73 g/100 g DW (Souid et al. 2021), and between 0.56 and 1.63 g/100 g DW (Generalić Mekinić et al. 2018) were reported in sea fennel harvested in Algeria, France, and Croatia, respectively. Chlorogenic acid is a hydroxycinnamic acid described in literature as one of the most widely distributed and functional polyphenols in the human diet, displaying health beneficial effects, behaving as antioxidant, anti-inflammatory, antimicrobial, antimutagenic, cardiovascular protective, neuroprotective, renoprotective, gastrointestinal tract-protective and hepatoprotective agent, and modulating lipid and glucose metabolism (Naveed et al. 2018; Lu et al. 2020).

## Conclusions

In conclusion, the characterization of constituents with potential pharmacological activity performed on sea fennel (*Crithmum maritimum*) cultivated in the Conero Natural Park highlighted similar polyphenolic profiles among different sprouts despite slight differences in the concentration of the single compounds, and confirmed the predominance of chlorogenic acid in the phenolic fraction. Moreover, the use of accelerated solvent extraction and a comprehensive characterization by HPLC-DAD-HRMS allowed the annotation of a wide range of phenolic constituents, some of them new for sea fennel, and triterpene saponins as well as hydroxylated fatty acids, again newly detected in this plant.
